# Tetrandrine and thapsigargin release arachidonic acid from cells in culture and stimulate prostacyclin production in rat liver cells, but may do so by different pathways

**DOI:** 10.1186/1471-2210-5-12

**Published:** 2005-06-24

**Authors:** Lawrence Levine

**Affiliations:** 1 Department of Biochemistry, Brandeis University, Waltham, MA 02454, USA

## Abstract

**Background:**

Tetrandrine inhibits tumor cell proliferation and demonstrates chemoprevention in cancer models. Speculation on the association between its effects on K^+ ^and Ca^2+ ^channels and cancer chemoprevention has been made. Thapsigargin also affects K^+ ^and Ca^2+ ^conductance. Thapsigargin, however, is a weak tumor promoter in the two-stage model of mouse skin carcinogenesis, yet it can induce apoptosis in androgen-independent prostatic cancer cells. I have postulated that arachidonic acid release from cells in culture is associated with cancer chemoprevention. The effects of tetrandrine and thapsigargin on arachidonic acid release from human colon carcinoma and rat liver cells and prostacyclin production by rat liver cells are compared in the current studies.

**Results:**

Tetrandrine and thapsigargin stimulate arachidonic acid release from human colon carcinoma and rat liver cells and prostacyclin production in rat liver cells. The stimulation by tetrandrine is not affected by incubation with actinomycin D, 100 mM KCl, the [Ca^2+^]_i _chelator, 1,2-*bi*s (*o*-amino-5-fluorophenoxy) ethane-*N,N,N',N'*,-tetraacetic acid tetraacetoxymethylester (BAPTA/AM) or in the absence of extracellular Ca^2+^. In contrast, stimulation by thapsigargin *is *inhibited by incubation with actinomycin D, 100 mM KCl, BAPTA/AM or in the absence of extracellular Ca^2+^.

**Conclusion:**

Both tetrandrine and thapsigargin stimulate arachidonic acid release, but based on the different results obtained in the presence of actinomycin D, the [Ca^2+^]_i _chelator, 100 mM KCl and in the absence of extracellular Ca^2+^, the mechanisms leading to this release and pathways leading to apoptosis and/or cancer chemoprevention may be different. Stimulations by tetrandrine may be mediated by activation of a secretory phospholipase A_2_, whereas thapsigargin's stimulations may be mediated by the cytoplasmic Ca^2+^-dependent phospholipase A_2_.

## Background

**Figure 1:**
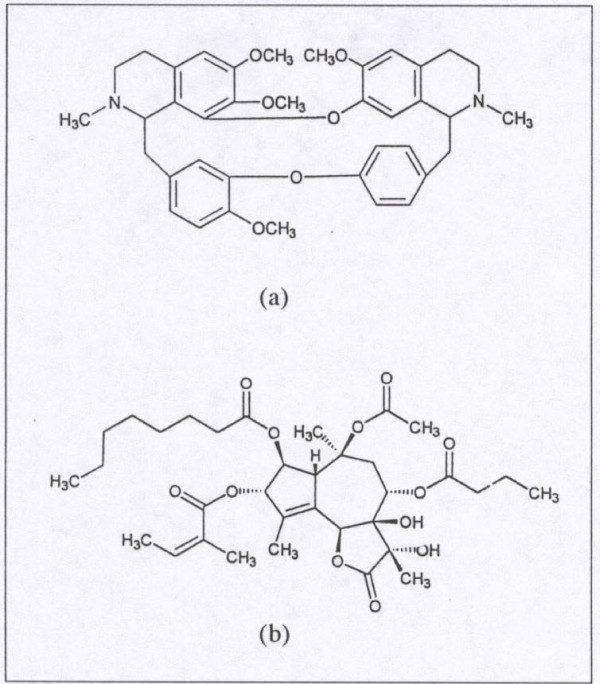
a): Tetrandrine (TET), isolated from the plant *Stephania tetrandra *(Structure reproduced with permission from G. Wang [9]) and b): Thapsigargin(THAP), isolated from the plant *Thapsia garganica *(Structure reproduced with permission from S. B. Christensen [15]).

Tetrandrine (TET), a bisbenzylisoquinoline (Fig. 1a), isolated from the root of the plant *Stephania tetrandra *has a number of potential medicinal properties. These include blockage of voltage-gated Ca^2+ ^channels [1], large-conductance Ca^2+ ^activated K^+ ^(BK) channels, and intracellular Ca^2+ ^pumps [1-6]. TET also has anti-inflammatory [2,7] and anti-cancer activities [8,9]. TET stimulates prostaglandin (PG)E_2 _production by macrophages [10], probably after first releasing the substrate, arachidonic acid (AA) by altering phospholipase (Plase) activities. TET also induces apoptosis in many cell types including human leukemic (U937), human lung carcinoma (A549), human hepatoblastoma (HEPG2), neuro 2a mouse neuroblastoma and rat glioma cells (C-6) [11-14].

Thapsigargin (THAP), a hexaoxygenated tetracycle sesquiterpine lactone, (Fig. 1b) isolated from the plant *Thapsia garganica*, also has a number of potential medicinal applications [15]. However, THAP is classified as a weak tumor promoter as measured in the two-stage model of mouse skin carcinogenesis [16]. Nevertheless, THAP [17] and its enzymatically modified analog [18] have been proposed as targeted therapy for prostate cancer. THAP, like TET, blocks intracellular calcium pumps resulting in increased cytoplasmic Ca^2+^, ([Ca^2+^]_i_) [reviewed in 15]. It also affects ion channels. THAP induces a Ca^2+^-dependent release of AA from [^3^H]-AA labelled macrophages and stimulates AA metabolism in the rat peritoneal macrophages [19]. THAP induces apoptosis in many cells including human neuroblastoma, colon cancer and prostate cancer cells and thymocytes [17,20-22].

Based on the stimulation of AA release by known cancer chemopreventative agents, I have proposed that AA release by cells is associated with cancer chemoprevention [23-27], possibly, but not necessarily, by activating a secreted tumor suppressor phospholipase A_2 _(PLA_2_) [28,29]. In this report, evidence is presented that TET, a potential cancer chemopreventive compound, and THAP, a weak tumor promoter that also possesses potential cancer preventative properties for androgen-independent prostate cancer, both stimulate AA release from human colon carcinoma and rat liver cells. Both compounds also stimulate prostacyclin (PGI_2_) production in rat liver cells. The release of AA and AA metabolites appears to be initiated by different mechanisms.

## Results

**Figure 2:**
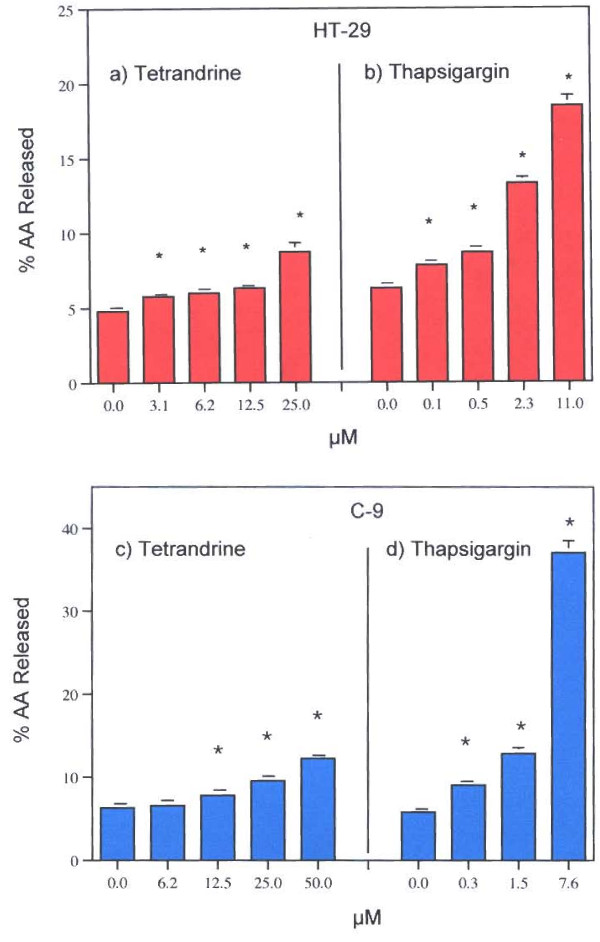
Effect of a) TET and b) THAP on AA release from HT-29 cells. Effect of c) TET and d) THAP on AA release from C-9 cells. The data are representative of several experiments. The analyses were performed with triplicate or quadruplicate dishes. * = Statistically significant *vs *MEM/BSA

TET and THAP release AA from human colon carcinoma (HT-29) cells and rat liver (C-9) cells in a concentration-dependent fashion (Fig. 2a – 2d respectively). As little as 0.1 to 0.3 μM THAP stimulates AA release. With both HT-29 and C-9 cells, THAP is about 10 to 30 times more potent than TET. Characterizations of these effects are shown in Table 1. Pre-incubation with actinomycin D partially inhibits stimulation by THAP but does not affect stimulation by TET. TET's stimulation of AA release does not require new mRNA synthesis, whereas THAP's stimulation does. As shown below, THAP's stimulation is mediated, in part, by the Ca^2+^-dependent PLA_2 _an induced enzyme. The absence of extracellular Ca^2+ ^partially inhibits THAP's stimulations but does not affect the AA release stimulated by TET. Depolarization of the cells with 100 mM KCl does not affect TET's stimulations, but does partially inhibit release of AA stimulated by THAP. Pre-incubation with the L-TYPE Ca^2+ ^channel blocker, diltiazem, has no effect on the actions of either TET or THAP in HT-29 or C-9 cells.

**Table 1:** Effects of Diltiazem (50 μg/ml), Actinomycin D (1 μg/ml), EGTA (1 mM), KCl (100 mM), and BABTA/AM (16 μg/ml) on AA Release from HT-29 or C-9 Cells stimulated by TET or THAP.

Some biological effects*	**Agent tested**	**Action of test agent**	**AA Release**	
			HT-29	C-9
**TET**	Diltiazem	Blocks L-type Ca^2+ ^channels	NI	NI
Ion Channels	Actinomycin D	Inhibits RNA synthesis	NI	NI
Apoptosis	EGTA	Chelates extracellular Ca^2+^	NI	NI
Depolarization	100 mM KCl	Depolarizes	NI	NI
[Ca^2+^]_I_	BAPTA/AM	Chelates [Ca^2+^]_i_	NI	**

**THAP**	Diltiazem	Blocks L-type Ca^2+ ^channels	NI	NI
Ion Channels	Actinomycin D	Inhibits RNA synthesis	↓	↓
Apoptosis	EGTA	Chelates extracellular Ca^2+^	↓	↓
Depolarization	100 mM KCl	Depolarizes	↓	↓
[Ca^2+^]_i_	BAPTA/AM	Chelates [Ca^2+^]_I_	↓	**

* = References in text

NI = No Inhibition (or stimulation)

↓ = Inhibition: statistically significant

** = BAPTA/AM (16 μg/ml) stimulates AA release (6.17 ± 0.088 (4)), MEM/BSA control *vs *(11.6 ± 0.322 (4)), BAPTA/AM (16 μg/ml). Thus, the effect of BAPTA/AM on C-9 cells is not recorded.

**Figure 3:**
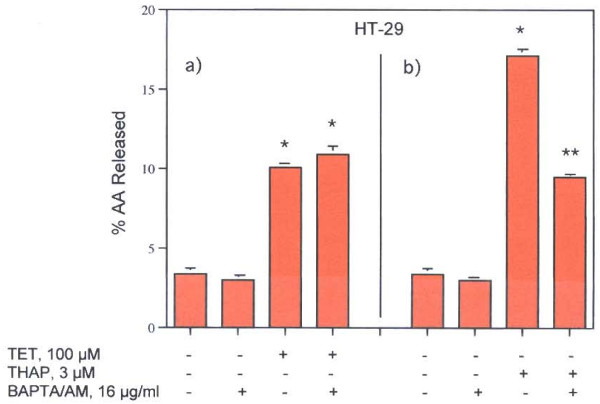
Effect of BAPTA/AM, 16 μg/ml, on AA release by a) TET and b) THAP from HT-29 cells. The data are representative of two experiments, each with similar results. The analyses were performed on quadruplicate dishes. * = Statistically significant *vs *MEM/BSA.

**Figure 4:**
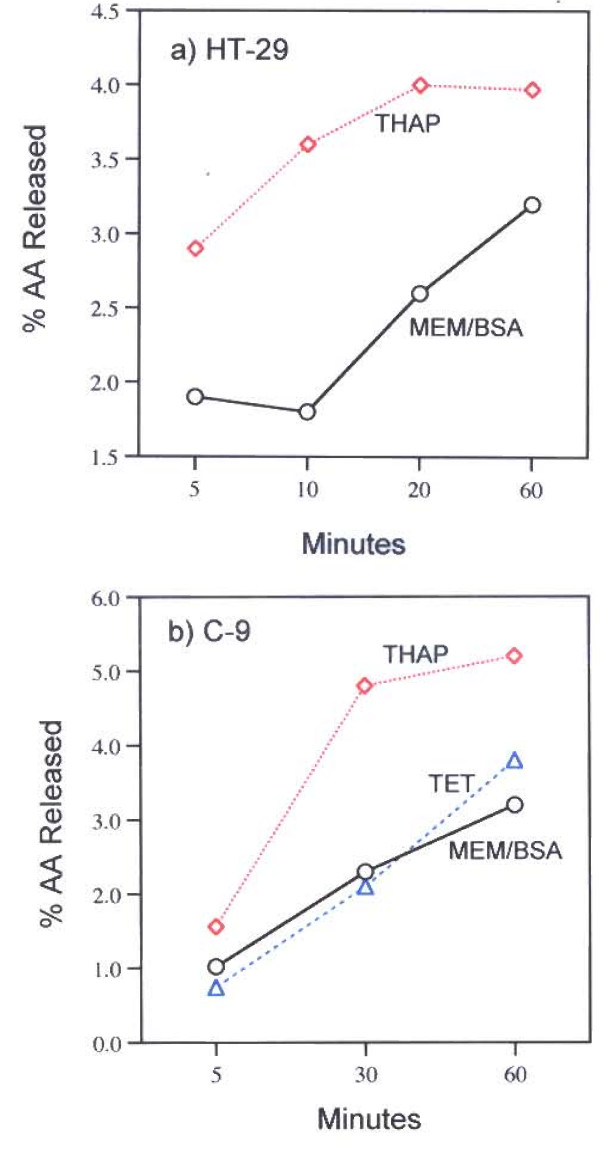
Time-course of AA release from a) HT-29 and b) C-9 cells by TET (50 μM) and THAP (2 μM). The analyses were performed on duplicate dishes. After the 60 minutes incubation, the AA release by TET is not statistically significant. TET's stimulation of AA release from HT-29 cells was not done.

The role of [Ca^2+^]_i _in these stimulations is striking. While pre-incubation with the [Ca^2+^]_i _chelator, BAPTA/AM (16 μg/ml), does not affect TET's stimulation of AA release from HT-29 cells, such treatment does inhibit THAP's stimulation of AA release from the HT-29 cells (Fig. 3, Table 1). Thus, a role for intracellular Ca^2+ ^pumps is strongly suggested only for the action of THAP. Ohuchi *et al *[19] had shown that [Ca^2+^]_i _increased within 2 min after treatment with THAP as measured by fluorescence changes in Quin 2 loaded peritoneal cells. A similar rise in [Ca^2+^]_i _has been measured after administration of THAP to thymocytes, rat liver microsomes [30] and in macrophages, astrocytoma cells, fibroblasts and human tumor lymphocytes [31]. The findings that the increased AA release is stimulated 5 minutes after incubation of HT-29 and C-9 cells with THAP is consistent with a role for increased [Ca^2+^]_i _in the activities of THAP (Fig 4a, 4b).

**Figure 5:**
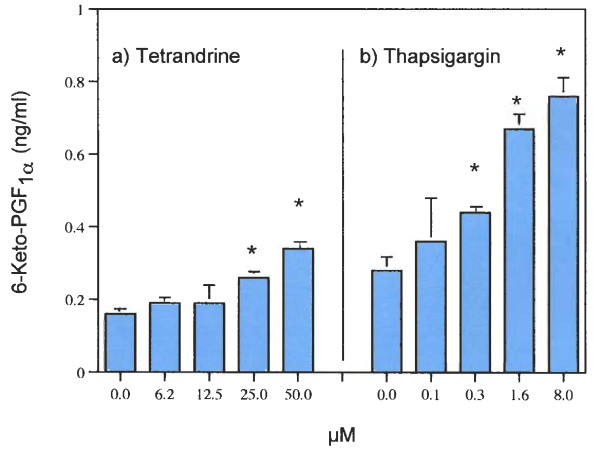
Effects of a) TET and b) THAP on PGI_2 _(6-keto-PGF_1α_) production in rat liver cells (C-9). The data are representative of several experiments. The analyses were performed with triplicate dishes. * = Statistically significant *vs *MEM/BSA.

While both cells express COX activity [32,33], only the major product of COX activity (PGI_2_) can be quantitatively estimated at the low cell densities used in these studies. Both TET and THAP stimulate PGI_2 _(measured as 6-keto PGF_1α_) production in these C-9 cells (Fig. 5a, 5b). As with AA release, THAP is at least 10-times more potent at stimulating PGI_2 _production than TET.

**Figure 6:**
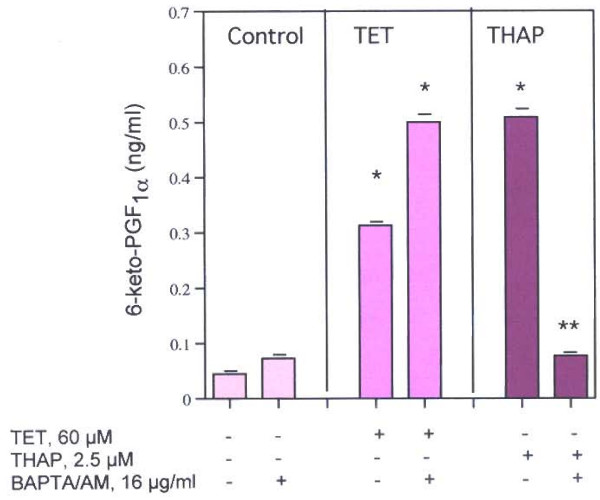
Effect of BAPTA/AM, 16 μg/ml on THAP's stimulated PGI_2 _production by rat liver cells. The data are representative of two experiments, each with similar results. * = Statistically significant *vs *THAP or BAPTA/AM. ** = Statistically significant *vs *THAP.

The likely role of [Ca^2+^]_i _in the stimulation of PGI_2 _production by THAP is shown in Fig. 6. Chelation of the increased [Ca^2+^]_i _with BAPTA/AM completely inhibits the stimulation by THAP but has no effect on the stimulation with TET.

## Discussion

It has been proposed that the cancer chemopreventative properties attributed to TET reside in its ability to effect ion channels which leads to inhibition of cell proliferation and apoptosis [9]. Retinoic acids, tamoxifen, PPAR-agonists, (e.g GW-7845), some non-steroidal anti-inflammatory drugs, vitamin D_3_, anti-oxidants, (e.g resveratrol and caffeic acid phenylester) and statins all release AA from cells in culture [23-26]. All have been shown to have cancer preventative properties. Results of studies on correlation, if any, of AA release and cancer chemoprevention by TET or THAP have not been published. For the purpose of this study, however, I have postulated that the cancer chemopreventive properties of the test agent are causally related to the capacity of that ligand to release AA. I have also suggested that most, if not all, of these agents at μM concentrations, may be intercalating into the cell membrane and releasing AA as a result of activated PLAse activity [25,26].

The mechanism of action of non-steroidal anti-inflammatory drugs that leads to apoptosis and cancer chemoprevention also involves stimulation of AA release and is associated with sphingomyelin to ceramide conversion [34]. The AA release probably results in ceramide-mediated apoptosis [35]. Both TET and THAP stimulate the release of AA from human colon carcinoma and rat liver cells. TET does prevent cancer [reviewed in 8] but THAP is considered to be a weak tumor promotor as measured in the two-stage model of mouse skin carcinogenesis [19]. THAP and some of its analogs, however, do induce apoptosis in androgen-independent prostatic cancer cells [17,18,36]. Apoptotic effects of THAP also have been reported in thymocytes and mouse lymphomas, including the WRH17-2 and WHB12 cell lines [17,20-22].

Clear differences in the pathways of AA release stimulated by TET and THAP were found in this study. TET stimulates AA release (Fig. 2) in HT-29 and C-9 cells and PGI_2 _production in C-9 cells (Fig. 5). It has been reported that TET blocks voltage-gated Ca^2+ ^channels [1-7] and depolarizes the cells [8], but blockage of these channels does not affect AA release (Table 1). Nor is the AA release stimulated by TET blocked by pre-incubation of the cells with actinomycin D, in the presence of 100 mM KCl or in the absence of extracellular Ca^2+^. It is interesting to note that cycloheximide (178 μM), did not affect TET's induced apoptosis in human lymphoblasts (CEM-C7) [37]. TET's stimulation of AA release is not affected by the [Ca^2+^] chelator, BAPTA/AM (Fig. 3). In contrast, THAP's release of AA by HT-29 cells is blocked by BAPTA/AM (Fig. 3), is inhibited by pre-incubation with actinomycin D, is inhibited by EGTA and is inhibited when the cells are incubated in 100 m M KCl (Table 1). That the rise in [Ca^2+^]_i _resulting from treatment with THAP appears to be associated with the stimulation of AA release is suggested by the relatively early increase, after 5 minutes, of AA release (Fig. 4). Both TET and THAP stimulate PGI_2 _production in the C-9 cells (Fig. 5), but only the PGI_2 _production stimulated by THAP is inhibited by BAPTA/AM (Fig 6).

The relationship between AA release and cancer chemoprevention by TET and THAP could be explained, in part, by implicating the PLA_2 _enzymes that catalyze the release of AA from phospholipids. The tumor suppressing sPLA_2 _may be only one of sixteen structurally different PLA_2 _enzymes [38,39]. If the tumor suppressor genes are overexpressed or activated, at least two cancer preventative pathways may result, one from the activity of the group 11A tumor suppressor [Reviewed in [39]] and a second from the AA released. The proximity of the PLA_2 _to the AA-esterified phospholipid after treatment of the cells is of importance, and may depend on the location of binding of the test agent. For example, celecoxib binds at the upper hydrocarbon core, close to the phospholipid head groups whereas rofeco

xib binds at the polar head groups of the membrane [40]. Celecoxib and rofecoxib differ in their release of AA [41]. TET may activate a tumor suppressing sPLA_2_, while THAP induces the Ca^2+^-dependent cPLA_2_. Both would lead to AA release.

## Conclusion

Cells treated with tetrandrine and thapsigargin share several common features including pathways that lead to induction of apoptosis. These properties probably reflect their interaction with cell membranes and the altered expression of signaling processes. One reaction that does not appear to be shared is deesterification of a phospholipid by a PLA_2_. Based on the effects of inhibition of *m*RNA synthesis, 100 mM KCl, extracellular and especially intracellular Ca ^2+^, thapsigargin's release is mediated, in part, by a Ca ^2+^-dependent PLA_2_, whereas tetrandrine's stimulated release is mediated by a secretory PLA_2_. In addition to the altered signaling properties that accompany the membrane intercalation, both tetrandrine and thapsigargin release biologically active AA.

## Methods

The rat liver (C-9 cell line) were purchased from the American Type Culture Collection (Manassas, VA, USA) and the human colon carcinoma (the HT-29 cell line) was obtained from Dr. Basil Rigas, Chief, Division of Cancer Prevention, SUNY at Stony Brook, NY, USA. They were maintained in Eagle's minimum essential medium (MEM) supplemented with 10% fetal bovine serum. [^3^H] AA (91.8 Ci/mmol) was obtained from NEN Life Science Products, Inc. (Boston, MA, USA). BAPTA/AM, the [Ca^2+^]_i _chelator, diltiazem, the L-type Ca^2+ ^channel agonist, tetrandrine and thapsigargin were purchased from BIOMOL International, (Plymouth, PA, USA). All other reagents were from Sigma Chemical Co. (St. Louis, MO, USA) or Calbiochem, (San Diego, CA, USA).

Two days prior to experiments, the HT-29 or C-9 cells were treated with 0.25% trypsin-EDTA and, after addition of minimal essential media (MEM) containing 10% fetal calf serum, the floating cells were seeded on to 35 mm culture dishes. The plating densities varied from 0.1 to 0.5 × 10^5 ^cells/35 mm dish. The freshly seeded cultures were incubated for 24-h to allow for cell attachment. After decantation of MEM containing the fetal bovine serum, 1.0 ml fresh MEM containing 10% fetal bovine serum and [^3^H] AA (0.2 mCi/ml) were added and the cells incubated for another 24-h. The cells were washed 4 times with MEM and incubated for various periods of time with 1.0 ml of MEM containing 1.0 mg BSA/ml (MEM/BSA) and different concentrations of each compound. The culture fluids were then decanted, centrifuged at 2000 × g for 10 min, and 200 μl of the supernate counted for radioactivity. The MEM/BSA values are the control values. Radioactivity recovered in the washes before the incubation was compared to input radioactivity to calculate the % radioactivity incorporated into the cells. For experiments on the effects of actinomycin D, the HT-29 or C-9 cells were washed with MEM 4 times, incubated with actinomycin D (1 μM) for 2-h, washed once and incubated again for 6-h in the presence or absence of actinomycin D (1 μM). For the effects of BAPTA/AM, the pre-incubation was 50 minutes. For PGI_2 _production, 1.0 ml of MEM supplemented with 10% fetal bovine serum, void of [^3^H] AA, was added after the first 24-h incubation. The cells were incubated for another 24-h, washed three times with MEM, then incubated with the compounds in MEM/BSA for various periods of time. The culture fluids were decanted and analyzed for 6-keto-PGF_1α_, the stable hydrolytic product of PGI_2_, by radioimmunoassay [42].

The [^3^H] AA release is presented as a percentage of the radioactivity incorporated by the cells. Except for the time-course experiments, which used duplicate dishes, three to five culture dishes were used for each experimental point. The data are expressed as mean values ± SEM. The data were evaluated statistically by the unpaired *Student's t-test*. A *P *value < 0.05 was considered significant.
